# The filter bubble and its effect on online personal health information

**DOI:** 10.3325/cmj.2016.57.298

**Published:** 2016-06

**Authors:** Harald Holone

**Affiliations:** Faculty of Computer Sciences, Østfold University College, Østfold, Norway

Search engines and social networks are increasingly used for health related inquiry by the public. The information found through search engines, or presented by social network services are typically tailored to the individual through the use of complex algorithms taking into consideration comprehensive information about the individual performing the search, often without the knowledge of the searcher. In this paper, I discuss how the technology poses challenges both for patients and clinicians, and present some ideas to mitigate these problems.

## A scenario

Imagine sitting in front of your computer trying to decide whether or not your children are to be vaccinated against common childhood diseases. You go to Google Search, and search for “vaccines and children”. You will get an overwhelming number of results (at the time of writing, I got about 35 million hits). The results are sorted and presented to you, with the ten top hits on the first page. Most people will click one of the links on that first page. What’s interesting is that the results are sorted not only by objective relevance, but rather is heavily influenced by your search history, your social network, when you are searching, and where you are searching from. In fact, over 200 so called “signals” go into that simple search, making your results almost certainly different from mine.

In most cases, this personalized search is beneficial to us, since it produces results that seem relevant to the user. However, as I will argue in this paper, there are serious problems with this, which in certain situations can mean the difference between life and death. In our example, it could mean the difference between choosing to vaccinate a child, or leaving it vulnerable to common, easily preventable diseases. The main reason why this may happen is that the technology we are using is hiding the complexity of the search algorithms, and is not revealing the additional information on which the filtering is based.

This is a problem for at least two reasons. First, most people do not know about this filtering, and even if they do, it is still inherently difficult to understand and grasp how it influences the search results. Second, the way the algorithms work can lead to the creation of a filter bubble (1), to use Eli Parisier’s term. The aim of this paper is to shed light on the effects of the filter bubble on online personal health information.

## The filter bubble

In 2011, Eli Parisier released The Filter Bubble: What the Internet Is Hiding From You (1). In this book, Parisier explains how the internet search engines and their algorithms are creating a situation where users increasingly are getting information that confirms their prior beliefs. Search algorithms are using large quantities of information about the user to find and present relevant information to the individual user. Your search and browse history is a key piece of the information used to tailor the results you get when you perform online searches. Combining this with information about your social network, viewing habits and geography leads to an increasingly narrow view on the information available online.

Parisier’s main argument is that this narrowing creates a filter bubble, which is invisible to the user, but still has immense impact on the information available to the individual.

When you perform a Google search, the information about you is used in addition to your search term to find and prioritize the search results most likely to be of your interest. Then, when you click among the first search results (as most people do), you are confirming back to the search engine that the results were indeed relevant and/or interesting. This in turn strengthens the filter, making it more likely that you will receive similar results in the future.

However, it is not only your own behavior that influences the results. The interests and preferences among people in your social network are also part of the algorithms, making it more likely that you will receive search results that your social network in general is gravitating toward. In many cases, these filters are providing relevant and good results. However, it becomes a problem as soon as your profile contains elements that make the search results gravitate toward misinformation.

The filters are to a large degree invisible, which adds to the problem. Many users are not even aware that the filtering is taking place, and even if they are, it is difficult to take control of how the filter is being applied. Granted, you can go to Google and delete your search history, or click the “Hide private results” button in the top right of the search results. Still, the complexity of the algorithms and the lack of usable explanations about how the filters actually work make it difficult for the user to take control.

The way the filters influence search results have led our group to use the term Gravitational Black Holes of Information to illustrate how difficult it is to break out of the force of the filters. As soon as you are aiming in at a core of misinformation, it is inherently difficult to break out of the gravitational force of the search algorithms. On the way toward the gravitational center, your prior believes are being strengthened by the new information you find, further pulling you into the black hole.

Naturally, the technology is not solely responsible for the quality of the information we find. Our prior beliefs, and the sources we seek for information are personal starting points that influence how we approach the information gathering. However, as the search algorithms learn about our preferences and history, the personal starting points are embedded into the technology as part of the filter algorithms.

Having introduced the filter bubble, I now describe the use of social media and internet search for health information, before looking at the filter bubble in a health context.

## The internet’s role in health information to the public

One of the aims of the Knowledge Landscapes network is to better understand how the public uses online resources to make decisions about personal health (2). The rise of the internet as a common medium has led to well documented changes in how people in general get informed about their own health situation (3,4). The commonly called “Doctor Google” is used from everything from self-diagnosis to information about drugs, epidemic outbreaks (5) and possible treatments for medical conditions. However, not only the big search engines, such as Google and Bing, are used for this kind of knowledge seeking. People use social networks such as Facebook, its groups, and pages to find like minded people or others in similar health conditions. On top of this, there’s a multitude of other forums online where people meet, read, discuss, and learn.

Access to this vast amount of information and resources changes the relationship between patients and doctors (6). The patients are often better prepared before their doctor appointments. The doctor used to be the medical authority, however studies show that the dynamics in the relationship between patient and doctor is changing due to the use of online resources (7). This poses new challenges for the doctors as well, perhaps spending less time informing the patient about basic information, and engaging more in medical discussions directly with the patient.

Of course, internet resources are also valuable tools for the doctors. Recently, on Norwegian TV, a show called “What’s Wrong with You?” lets three skilled medical doctors and three members of the public compete to diagnose a variety of patients. The public team is using internet search as a tool, whereas the medical doctors are only allowed to use their joint knowledge, no external resources allowed. Despite being rigged for entertainment purposes, the show is a good illustration of the new dynamic between the public and the medical authorities. Given that the increased use of online sources for health information has implications on the choices people make about their own health, it becomes important to look at how the filter bubble can play an important, possibly dangerous role in the type and quality of health information people are accessing online.

## Effects of the filter bubble on health information search

Returning to the vaccination scenario from the introduction, it becomes obvious that the search history, social network, personal preferences, geography, and a number of other factors influence the information found by the searcher. In this particular case, the decision about whether or not to vaccinate the children can, to a large degree, be driven by the filter bubble. This is not a made-up example. In 2014, 23 measles outbreaks and more than 644 cases of measles were reported in the US (8). Perhaps most famous is the 2014/2015 outbreak in Disneyland in California. One of the reasons for the outbreak was a growing concern among parents about the efficiency and side effects of vaccination. Certain anti-vaccine organizations and high-profile individuals have been successful in disseminating misinformation and fear, contributing to a lowered vaccination rate, leading to an increased number of people catching the easily preventable measles disease. Vaccine information is only one example where the filter bubble contributes to the spread of misinformation. The problem applies to all areas of health information; from diets and nutrition, to cancer treatments and epidemic outbreaks (5). Where representatives of the medical profession have to take precautions and necessarily be vague in their communication with the public, the problem increases. Opposing powerful anecdotes from individuals, eg, for perceived successful alternative cancer treatments, the medical society struggles to get through with their more balanced and scientific message. Further, when there are internal discussions in the medical field concerning relevant diagnosis and treatments, the public in search of answers can be even more susceptible to misinformation. One example is Myalgic encephalomyelitis (ME), where the professionals disagree about whether the condition has somatic or psychological roots. Anecdotal information always has a strong appeal, and the appeal is strengthened when there is no “true” explanation, or a simple, quick fix for the condition.

Again, the public in search of an answer can be drawn toward the gravitational black hole of information, and be led to make unhealthy decisions for themselves or members of their family. Obviously, the filter bubble is not solely responsible for the wrong type of information reaching the public. Preconceived notions about the issues people are searching for influence the kind of information they find regardless of the filter bubble. However, the added effect of the filter bubble increases the challenge, often resulting in an even stronger conviction that the preconceived notions are correct. The filter bubble can lead to increased confirmation bias (9).

It is tempting to view the filter bubble as equivalent to an invisible in-car navigation system, which instead of suggesting the direction you should follow, simply takes control of your car and takes you where it thinks you want to go. An automation system that does not allow the user to take a pause and consider the effects of automation can lead to mis-use, frustration, and accidents (10).

In short, the main problem is not the search algorithms as such; they are welcome in most cases, and help us navigate extensive amounts of information in a manageable way. The problem is the invisibility of the algorithms, both in terms of the way they are hidden from explicit view on search engines and social networks, and the way they directly impact the quality of the information we find when we look for information online.

## Possible actions

The effects of the filter bubble in a health context is a complex problem area, and the solutions are not readily available. However, it is tempting to propose a few possible directions. **Information to the public** about the filter bubble, the hidden algorithms, and the effect it has on our online lives must be part of the solution. If the public (and the professionals) are unaware of the problem, the problem will continue to grow.

Providing the possibility of **unfiltered search**, allowing the public to get un-biased information based on relevance and content quality is one way forward. Google is providing this through their “hide private results” button on their search engine. However, it is still unclear how the remaining filter algorithm is working, and it seems that most people are unaware of the possibility. Many other services do not offer the same option.

People could **switch to another search engine or service**, such as duckduckgo.com. However, most people are loyal to their trusted brand (such as Google), and for other types of services (such as Facebook), the investment in a social network and the lack of alternative services make this difficult.

Online services could enable the public to **engage in sensemaking** (11). This requires presentation not only of the search results, but also information about how the search results were produced. Given the complexity of the filters, this poses a major challenge to the service providers when it comes to visualization of the search results.

## Conclusion

The filter bubble influences the way the public find personal health information online. The algorithms that support us in finding relevant information quickly can also bring us closer to a gravitational black hole of information, which subsequently can lead us to make bad decisions about health issues. This problem will not go away by itself, and I have suggested a few ways forward to help alleviate the problem. Hopefully, through the Knowledge Landscapes network, we will gain an even better understanding of the way the public use online resources for managing their own health, and be able to provide ideas that can improve the quality of health related information that reaches the public.
